# Biocatalytic Behaviour of Immobilized *Rhizopus oryzae* Lipase in the 1,3-Selective Ethanolysis of Sunflower Oil to Obtain a Biofuel Similar to Biodiesel

**DOI:** 10.3390/molecules190811419

**Published:** 2014-08-04

**Authors:** Carlos Luna, Cristóbal Verdugo, Enrique D. Sancho, Diego Luna, Juan Calero, Alejandro Posadillo, Felipa M. Bautista, Antonio A. Romero

**Affiliations:** 1 Department of Organic Chemistry, University of Cordoba, Campus de Rabanales, Ed. Marie Curie, Córdoba 14014, Spain; E-Mails: qo1lumad@uco.es (D.L.); p72camaj@uco.es (J.C.); qo1baruf@uco.es (F.M.B.); qo1rorea@uco.es (A.A.R.); 2 Crystallographic Studies Laboratory, Andalusian Institute of Earth Sciences, CSIC, Avda. Las Palmeras 4, Armilla 18100, Granada, Spain; E-Mail: cverdugoe@lec.csic.es; 3 Department of Microbiology, University of Córdoba, Campus de Rabanales, Ed. Severo Ochoa, Córdoba 14014, Spain; E-Mail: edsancho@uco.es; 4 Seneca Green Catalyst S.L., Campus de Rabanales, Córdoba 14014, Spain; E-Mail: seneca@uco.es

**Keywords:** biodiesel, immobilization, *Rhizopus oryzae* lipase, Biolipase-R, amorphous AlPO_4_/sepiolite, demineralized sepiolite, selective transesterification, monoglyceride, Ecodiesel, sunflower oil

## Abstract

A new biofuel similar to biodiesel was obtained in the 1,3-selective transesterification reaction of sunflower oil with ethanol using as biocatalyst a *Rhizopus oryzae* lipase (ROL) immobilized on Sepiolite, an inorganic support. The studied lipase was a low cost powdered enzyme preparation, Biolipase-R, from Biocon-Spain, a multipurpose additive used in food industry. In this respect, it is developed a study to optimize the immobilization procedure of these lipases on Sepiolite. Covalent immobilization was achieved by the development of an inorganic-organic hybrid linker formed by a functionalized hydrocarbon chain with a pendant benzaldehyde, bonded to the AlPO_4_ support surface. Thus, the covalent immobilization of lipases on amorphous AlPO_4_/sepiolite (20/80 wt %) support was evaluated by using two different linkers (*p*-hydroxybenzaldehyde and benzylamine-terephthalic aldehyde, respectively). Besides, the catalytic behavior of lipases after physical adsorption on the demineralized sepiolite  was also evaluated. Obtained results indicated that covalent immobilization with the *p*-hydroxybenzaldehyde linker gave the best biocatalytic behavior. Thus, this covalently immobilized lipase showed a remarkable stability as well as an excellent capacity of reutilization (more than five successive reuses) without a significant loss of its initial catalytic activity. This could allow a more efficient fabrication of biodiesel minimizing the glycerol waste production.

## 1. Introduction

The production of biofuels has become increasingly important in last decade to partially satisfy the future energetic demands in the transport sector [1,2,3]. In this respect, for the valorisation of triglycerides to allow their use as biofuels in current diesel engines, there are very diverse methods such as the direct use of triglycerides as microemulsions and emulsifications, however the transesterification of oils and fats with short chain alcohols is currently the most attractive and widely accepted methodology for biodiesel production [4]. This usually involves the use of homogeneous base catalysts operating under mild conditions and glycerol is always obtained as the main by-product through a stepwise process. Thus, in addition to the alkaline impurities that need to be removed in the conventional method, the accumulation of glycerol is the main drawback of this method, not only because it represents a lowering in the yield of the process, but also because this residual glycerol must be cleaned from the obtained biodiesel, to avoid breakdowns in Direct Injection (DI) engines. Consequently, several consecutive washing steps are applied, where actually a lot of water is spent to achieve the complete glycerol elimination from the crude biodiesel, before its use and commercialization.

To avoid the associated problems in the conventional process, a series of alternative methods are under investigation. They are all in fact based on producing some glycerol derivative in the same transesterification process together with the fatty acid esters constituting the conventional biodiesel. In this way the compulsory, complex and expensive additional separation process of glycerol is avoided, so that not only it the waste generation prevented, but also the process yield is increased. These novel methodologies, using different acyl acceptors instead of methanol in the transesterification process, are able to produce methyl esters of fatty acids together to glycerol derivative co-products, soluble in the mixed reaction products. The drawback of glycerol is mainly due to its hydrophilic nature that prevents its solubilisation in the lipophilic mix constituting biodiesel [5]. Thus, the transesterification reactions of triglycerides with dimethyl carbonate (DMC) [6], ethyl acetate [7] or methyl acetate [8] can generate a blend of three molecules of FAME or FAEE and one of glycerol carbonate (GC) or glycerol triacetate (triacetin). Consequently, the atom efficiency is also improved because the totality of the atoms involved in the reaction are part of the final blend. Besides, these combinations including glycerol derivative molecules, have suitable physicochemical properties to be employed as a biofuel [9] in conventional DI motors.

Lipases (triacylglycerol acylhydrolase, EC 3.1.1.3) are hydrolases that operate on carboxylic ester bonds. They are widespread in all organisms, where their natural physiological role is the hydrolysis of triglycerides. However, they can also catalyze esterifications, alcoholysis and transesterifications in non-aqueous media [10]. Such versatility makes them ideal candidates for various applications in the food, detergent, pharmaceutical, leather, textile, cosmetic, and paper industries [11,12]. In this connection, in the last decade the enzymatic production of biodiesel has also been the subject of intensive research [13,14,15].

The pros and cons of using lipases as biocatalysts for biodiesel production compared to alkaline and acid catalysts are related to the short time and high yields obtained when chemical transesterification is applied. However, drawbacks such as high energy requirements, difficulties in the recovery of the catalyst and glycerol and potential environmental pollution are major disadvantages of the alkali- or acid-catalyzed processes [16,17]. In general, lipases perform their catalytic activity under more gentle conditions and with a variety of triglyceride substrates, including waste oils and fats with high levels of FFA. Furthermore, biodiesel separation and purification is much easier, resulting in a more environmentally friendly process [5,18]. However, the main bottleneck for the industrial application of lipases is their high cost. In this respect, several immobilization methods have been introduced to improve lipase stability and to allow its repeated utilization. By using this methodology a notable lowering in the cost impact of lipases could be obtained [19,20].

**Scheme 1:**
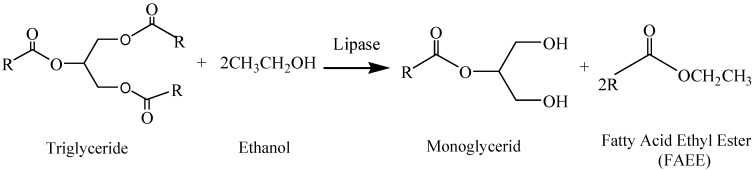
Representative scheme of the 1,3-selective partial ethanolysis of triglycerides obtained by using lipases as enzymatic biocatalysts. A biofuel with similar physicochemical properties to the conventional biodiesel is obtained, avoiding glycerol generation as byproduct and also increasing the reaction yield.

Most lipases have indeed a peculiar 1,3-regioselectivity, which means that they selectively hydrolyze the 1 and 3 positions in triglycerides [21]. In this regard, through the 1,3-selective partial ethanolysis of triglycerides with low cost lipases (like pig pancreatic lipase, PPL [22,23,24,25], or *Thermomyces lanuginosus* lipase (TLL) Lipopan 50 BG from Novozymes [26]), a reaction blend with two parts of FAEE and one part of MG it always obtained (Scheme 1), which integrates the glycerol as a monoglyceride, a soluble product in diesel fuel. In this way, the production of glycerol as by-product is avoided and the environmental impact of the process is also reduced. On the other hand, this biofuel that integrates glycerol as monoglyceride not only improves the atom efficiency of the process but also presents physicochemical properties quite similar to the conventional biodiesel obtained by alkaline catalysis, so it can be also employed as a biofuel [13] in conventional DI engines.

Accordingly, the enzymatic process to obtain this new biofuel operates under much smoother conditions, no impurities are produced, the biofuel obtained exhibits similar physicochemical properties to those of conventional biodiesel and monoacylglycerides (MG) enhance the biodiesel lubricity, as demonstrated by recent studies [27,28,29]. Moreover, the ethanol that is not spent in the enzymatic process also remains in the reaction mixture in such a way that, the product blend obtained after the reaction can be directly used as a viable fuel [30,31,32].

In regard to the presence of 33.3% of a monoglyceride (MG) in Ecodiesel, it is important to note that the physicochemical properties of an ester, whatever they may be, are completely different from those of the acids and alcohols from which it comes. In this respect, the glycerol works like a pollutant for the engine because it is an insoluble compound in biodiesel and/or conventional diesel, generating several problems in the running of diesel engines [27,28,29,30,31,32], so that there are strict limitations to control the presence of glycerol in any biofuel to be used in diesel engines. However, this problem does not happen with any of these molecules such as MG, DG or even TG, because they all are soluble in the blend of FAMEs that constitutes conventional biodiesel or in fossil fuel. Thus, the difference in physicochemical properties between a FAME molecule and a MG one are minimal, because both are esters and exhibit very similar molecular weights (the difference between methanol and glycerol) and in fact, FAEE exhibits molecular weights between FAME and MG. The EN 14214 and ASTM 6751 standards define the purity of a concrete biofuel, called biodiesel, but that does not mean that it is the only biofuel that can be used, pure or mixed with fossil diesel, in diesel engines. In this sense, besides Ecodiesel [3,22,23,24,25,26,33,34], there are different research lines targeting novel alternative biofuels similar to biodiesel that do not produce glycerol as by-product [3,4,5,6,7,8,9]. In each of these cases, glycerol is kept in the biofuel as its corresponding derivative, which is soluble with FAEE, FAME (conventional biodiesel) or fossil diesel.

The actual limitations of the use of lipases in industrial processes are currently associated with their high production costs. This can be overcome through the application of molecular technologies to achieve the production of purified enzymes in sufficiently high quantities as well as by the heterogeneous reuse of biocatalysts after the immobilization of lipases [19,20]. Thus, in order to obtain an effective, technically and economically viable enzymatic process, here the selective ethanolysis of triglycerides by the application of a biocatalyst constituted by immobilized Biolipase-R is reported. This is a low cost multipurpose additive from Biocon Spain used in food industry, where it is used as a powdered enzyme preparation, which contains *Rhizopus oryzae* lipase (ROL).

According to recent results [33], this powdered enzymatic preparation has shown a valuable biocatalytic behavior in the selective ethanolysis of sunflower oil, so that in order to improve this methodology, in the present research a study on the immobilized biocatalyst is developed. Thus, following a procedure previously described in the covalent immobilization of PPL [22,24,25,35], the current commercial biocatalyst is immobilized on amorphous AlPO_4_/sepiolite as inorganic support. In this respect, covalent immobilization of Biolipase-R on an inorganic support is carried out by using a previously described methodology for the heterogeneization of lipases [22,23,24,25,26], other enzymes like phosphatase [36] or glucose oxidase [37] as well as homogeneous organometallic complexes [38]. Besides, the selective alcoholysis of sunflower oil with ethanol obtained with Biolipase-R after physical adsorption on a demineralized sepiolite it is also evaluated [22].

Regarding the immobilization of lipases to allow the heterogeneous reuse of biocatalysts, covalent binding on the external surface of an insoluble support material is the most interesting enzyme immobilization methodology because in this way mass transfer limitations associated with other immobilization techniques, (entrapment or adsorption in gels) are decreased and it combines the high selectivity of enzymatic reactions with the chemical and mechanical properties of the support [39,40,41,42,43,44]. Nevertheless, most studies in this area have been developed on polymer-supported carriers because of the easy obtention of many different functional groups, to get efficient interactions with the enzymes. However, these organic supports suffer a number of problems such as a significant loss of stability of the catalytic material due to its swelling and poor stability towards organic solvent attack. This is especially true in the production of biodiesel through transesterification of triglycerides because of the high capacity of esters to dissolve and/or swell organic polymers. In this respect, inorganic supports such as silica, alumina, and layered double hydroxides, exhibit great physical strength and chemical inertness and are thermally and mechanically stable. Besides, inorganic solids also display an excellent storage stability for enzymes, so that the immobilization onto inorganic supports could thus improve the catalytic performance [24,25,35,36,37,38,39,40,41,42,43,44,45,46,47].

Amorphous or mesoporous silica is the support mainly used for covalent immobilization of lipases. Thus, micelle template silica constitutes a material with high thermal and mechanical stability, which can be modified and functionalized, during its synthesis by the sol-gel method, and by directly grafting some functional organosilane groups on silica surfaces [24,38]. However, the biocatalyst stability always will depend on the stability of the different organosilane bonds, which can be broken due to effects of the reaction conditions. In this respect, it is well known that organosilane bonds (Si-CH_2_-R) are very reactive, respect to hydroxyl groups in water or some alcohols, yielding a hydroxysilane (Si-OH) and the corresponding alkanes. Consequently, water or alcohol solutions, polar solvents, higher temperatures, *etc.*.. may promote the hydrolysis or alcoholysis of the organic–inorganic hybrid bonds, thus leading to varying degrees of enzyme leaching. On the contrary, in the procedure previously described for the covalent immobilization of lipases on inorganic supports [22,23,24,25,26,35,45,46,47], the organic–inorganic hybrid bonds are obtained through a phosphamide or phosphoester bond. In this way, more efficient links than organosilane bonds, between different organic molecules, including macromolecules like lipases, and the external surface of an amorphous inorganic solid such as AlPO_4_ are obtained. Thus, the high stability of phosphamide and phosphoester bonds plays a crucial role in the well-established structure of many different biomolecules, including RNA and DNA macromolecules. Thus, in the last years we have undertaken a series of research studies on the synthesis and characterization of different amorphous AlPO_4_, properly tailored by a controlled sol–gel method that allows us to obtain a high surface area as well as a high number of surface Brönsted acid sites, to facilitate the covalent attachment of lipases and other enzymes, as well as homogeneous organometallic complexes [22,23,24,25,26,35,36,37,38,45,46,47].

Thus, following this methodology, PPL was covalently immobilized on AlPO_4_/sepiolite support [24] with high yield (75 wt %), although the efficiency of the immobilized enzyme was reduced to approximately half (49.1%) compared to that of the free PPL. However, the covalently immobilized enzymes showed a remarkable stability as well as a great reusability (more than 40 successive reuses) without a significant loss of their initial catalytic activity. In this way, the life of this immobilized PPL is expanded in an extraordinary way from no more than 48 h with free PPL to months with successive reuse. Such a high number of reuses is not usually described in the literature with organic polymer supports or with inorganic supports activated with organosilane linkers [24,38]. This indicates the capability of the phosphamide bond to create efficient links between different organic molecules and the external surface of an amorphous inorganic solid such as AlPO_4_. This external AlPO_4_ layer works like an activating agent for the true inorganic support, the sepiolite. Thus, the high stability of phosphamide bonds plays a crucial role in the covalent immobilization of PPL biomolecules.

Taking into account the excellent previous results achieved in the immobilization of these enzymes [22,24,25], in this study we now have extended the possibilities of this methodology to be used to the covalent and physical attachment of Biolipase-R. In this way, to be applied as an economically viable biocatalyst for the production of a novel biofuel integrating glycerol into its composition as MG together to the different FAEEs in the enzymatic ethanolysis process as well as with the excess of unreacted ethanol. This biofuel mixture, currently named Ecodiesel [3,22,23,24,25,26,33,34] is able to directly operate diesel engines, alone or in whichever mixture with diesel fuel, without any further separation or purification.

In summary this research atttempts to add value to the process of selective ethanolysis of triglycerides with a low cost lipase [3,22,23,24,25,26,33,34] by application of heterogeneous catalysis after its immobilization on sepiolite, a natural hydrated magnesium silicate of fibrous nature, with a high surface area and low price. Accordingly, two covalent immobilization methods are comparatively evaluated regarding a method of physical immobilization of lipases, always using sepiolite as inorganic support.

## 2. Results and Discussion

### 2.1. Efficiency of Different Lipase Immobilization Procedures

**Figure 1:**
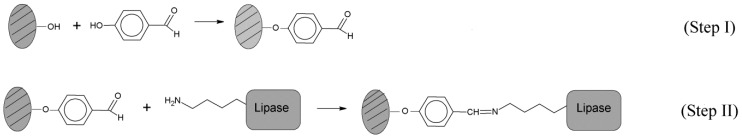
General scheme for the immobilization of the enzyme through the ε-amino groups of lysine residues. Amorphous AlPO_4_ activation is performed by microwave, heated with p-hydroxybenzaldehyde (**Step 1**) before to the covalent immobilization of the enzyme through lysine residues (**Step 2**).

**Figure 2:**
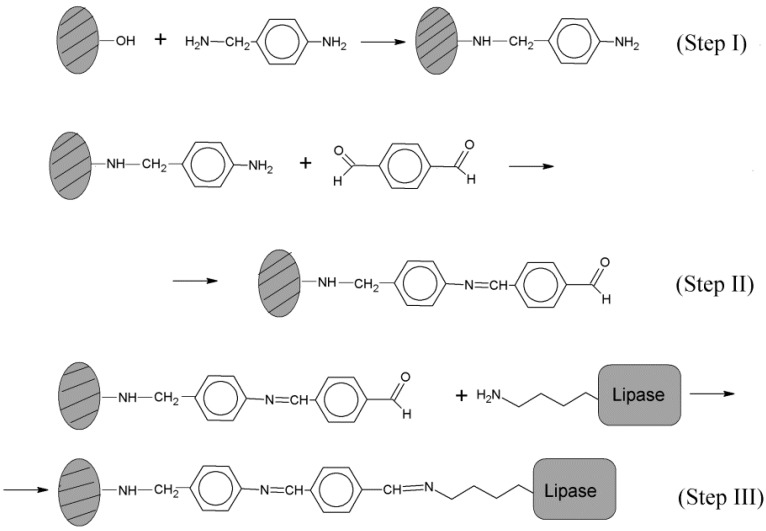
General scheme of the immobilization of *Rhizopus oryzae* lipase (ROL) through the ε-amino group of lysine residues to an organic linker bonded to the inorganic support. (**a**) In Step 1, surface OH groups of the supported AlPO_4_/Sepiolite are activated by microwave heating with 4-aminobenzylamine; (**b**) In Step 2, terephtaldicarboxaldehyde is reacted through imines bonds also obtained by microwave heating; (**c**) In Step 3 the covalent immobilization of the enzyme is obtained through the imines bonds produced with lysine residues.

In both covalent immobilization methods applied (Figure 1 and Figure 2, respectively), the key process is the formation of an imine bond between the organic linker bound to the inorganic support, and the residual amine group of the ε-amino groups of lysine in the protein constituting the lipases. This covalent immobilization process of proteins is carried out in pure absolute ethanol (99.9%) as an organic solvent, a neutral molecule, where the nucleophilic attack at the carbonyl group by the pair of unshared electrons of the nitrogen atom of any alkylamine occurs very easily, generating a tetrahedral intermediate (process of nucleophilic addition) followed by the removal of a water molecule. The latter process is promoted by the use of absolute ethanol, a substance with high affinity for water. Thus, an imine bond is irreversibly obtained. However, in aqueous media, or just with a small proportion of water, the imine bond will not be enough stable to allow an effective immobilization process.

Suitable results were achieved with the two different methodologies followed to obtain the support functionalization, in the covalent immobilization of Biolipase-R on AlPO_4_/sepiolite system (20–80 wt %) used as support. However, they have a distinct catalytic performance depending on the method used to achieve the covalent immobilization of lipases. Thus, the results shown in Table 1 and Table 2 display the importance of pH working conditions to get the lipase attachment, on catalytic behavior, as a function of the linker applied for the lipase immobilization.

**Table 1:** Percentage of immobilized enzyme, E_imm_, residual activity, E_res_, and specific activity of the immobilized enzyme, E_spe_, determined from the corresponding values of catalytic activity (mmol/min) of the free native enzyme, r_nat_, the activity of the filtrate in the immobilization process, r_fil_, and the activity of the immobilized enzyme, r_imm_, for the linker *p*-hydroxybenzaldehyde. The lipase immobilization has been carried out at pH 7 and 9 and at 30 °C.

pH	r_nat_ 10^2^ (mmol/min)	r_fil_ 10^2^ (mmol/min)	r_imm_ 10^2^ (mmol/min)	E_imm_ (%)	E_res_ (%)	E_spe_ (%)
7	14.28	2.9	1.8	79.7	12.6	15.8
9	12.5	10.0	9.0	20.0	72.0	360.0

Here the reaction rates for the immobilized system obtained using the *p*-hydroxybenzaldehyde linker (Table 1) as well as those achieved using a longer linker (Table 2), which was synthesized by the successive reaction of 4-aminobenzylamine and terephthalic aldehyde, according to the reaction schemes indicated in Figure 1 and Figure 2, respectively, can be seen. Thus, with the shorter linker *p*-hydroxybenzaldehyde (Figure 1) a substantial influence of the medium pH on the immobilization of the lipases is observed, so that at pH 7 a 79.7% lipase immobilization is achieved while it is only 20.0% at pH 9. However, the quality of the lipases immobilized at pH 7 is markedly lower than that obtained at pH 9, where the residual activity, E_res_, and the specific activity, E_spe_, of immobilized enzymes are significantly higher.

**Table 2:** Percentage of immobilized enzyme, E_imm_, residual activity, E_res_, and specific activity of the immobilized enzyme, E_spe_, determined from the corresponding values of catalytic activity (mmol/min) of the free native enzyme, r_nat_, the activity of the filtrate in the immobilization process, r_fil_, and the activity of the immobilized enzyme, r_imm_, for the linker obtained by reaction of 4-aminobenzylamine and terephtaldialdehyde. The lipase immobilization has been carried out at pH 7 and 9 and at 30 °C.

pH	r_nat_ 10^2^ (mmol/min)	r_fil_ 10^2^ (mmol/min)	r_imm_ 10^2^ (mmol/min)	E_imm_ (%)	E_res_ (%)	E_spe_ (%)
7	14.3	12.8	4.6	10.4	32.2	310.8
9	12.5	7.0	5.9	44.0	47.2	107.0

**Figure 3:**
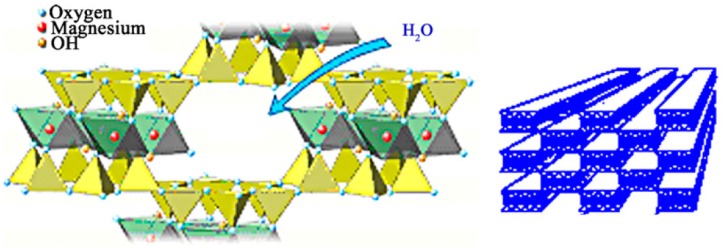
Structure of hydrated natural sepiolite.

However, when performed with a longer linker, as in the case of 4-aminobenzylamine bound to terephthalic aldehyde (Figure 3), the reverse happpens, because a higher amount of immobilized lipase is obtained when the immobilization is performed at a higher pH (9), also with higher residual activity, E_res_, although with less specific activity, E_spe_.

In both cases, covalent immobilization of the enzyme is achieved by the arrangement of an inorganic-organic hybrid composed by a functionalized hydrocarbon chain with pendant benzaldehyde, linked to the same inorganic support. Sepiolite is a natural silicate, which is activated on its surface by amorphous AlPO_4_. The functionalization is carried out on the surface hydroxyl groups of acidic character where the binding of the two studied linkers occurs by forming phosphoester bonds (Figure 1) or phosphamides (Figure 2), respectively. Thus, it appears that a differential effect is obtained due to the different length of the linkers, which allowa a different structural position of the immobilized lipases. In this respect, it is assumed that lipases after immobilisation may change their biocatalytic activity (increasing or decreasing to a variable extent). These changes seem to be related with the protein conformation obtained in the immobilization process. The experimental conditions under which the immobilization process is performed significantly affects the final conformation of the lipase after immobilisation. Therefore, the experimental results collected in Table 1 and Table 2 indicate that the pH conditions are able to affect the ability to immobilize the lipase as well as the specific activity of immobilized lipases.

In any case, according to the similar results obtained with both methods when they are applied operating under the best experimental conditions, the comparison between both systems of functionalization seems to advise the use of *p*-hydroxybenzaldehyde as activating agent of the inorganic solid, operating at pH 9. This method is comparatively the simplest, because the functionalization step is carried out in one step (Figure 1), compared to the two steps required with the process using 4-aminobenzylamine and terephthalic aldehyde to get the longer organic linker (Figure 2).

The immobilization of enzymes has also been carried out by physical adsorption of Biolipase-R in the three-dimensional porous structure of sepiolites, after being subjected to an acid treatment in order to clean the hydrated channels (Figure 3) of the different metal hydroxides like Al, Fe, alkaline ions and mainly Mg [35,36]. These channels can be filled with the lipases, producing in this way its immobilization by physical adsorption. Results obtained are collected in Table 3, where the influence of pH conditions on the physical adsorption of lipases is also pointed out. According to the results, contrary to what occurs in covalent immobilization, here an important influence of the pH on the physical adsorption process is not observed. Accordingly, at pH 7 the attachment of the 58.5% of the lipases is achieved, *versus* the 66.9% obtained at pH 9, so that the amount of immobilized lipase at pH 7 is not clearly lower than that obtained at pH 9. The residual activity, E_res_, and specific, E_spe_, of immobilized enzymes are also slightly higher at the higher pH 9.

**Table 3:** Percentage of immobilized enzyme, E_imm_, residual activity, E_res_, and specific activity of the immobilized enzyme, E_spe_, determined from the corresponding values of catalytic activity (mmol/min) of the native enzyme, r_nat_, the activity of the filtrate in the immobilization process, r_fil_, and the activity of the immobilized enzyme, r_imm_, for the immobilized lipase on demineralized sepiolite. The immobilization was carried out at pH 7and 9.

pH	r_nat_ 10^2^ (mmol/min)	r_fil_ 10^2^ (mmol/min)	r_imm_ 10^2^ (mmol/min)	E_imm_ (%)	E_res_ (%)	E_spe_ (%)
7	14.7	6.1	7.8	58.5	53.0	90.6
9	14.2	4.7	9.2	66.9	64.8	96.9

To achieve physical adsorption of lipases on sepiolite a previous demineralization treatment in acid medium is essential. This is clearly concluded from the results collected in Table 4 where sepiolite was used as a support without any previous treatment, before the lipase immobilization. Here it can be seen that at both studied pH values, the amount of immobilized lipase is practically negligible.

**Table 4:** Percentage of immobilized enzyme, E_imm_, residual activity, E_res_, and specific activity of the immobilized enzyme, E_spe_, determined from the corresponding values of catalytic activity (mmol/min) of the native enzyme, r_nat_, the activity of the filtrate in the immobilization process, r_fil_, and the activity of the immobilized enzyme, r_imm_, for the immobilized lipase on undemineralized sepiolite. The immobilization was carried out at pH 7 and 9.

pH	r_nat_ 10^2^ (mmol/min)	r_fil_ 10^2^ (mmol/min)	r_imm_ 10^2^ (mmol/min)	E_imm_ (%)	E_res_ (%)	E_spe_ (%)
7	14.1	12.8	0.6	9.2	4.3	46.7
9	14.5	13.5	1.2	6.9	8.3	120.3

On the other hand, it can be concluded that the best residual activity, E_res_, in all cases is obtained with immobilization at pH 9. This is the most important parameter from a practical point of view, because determines the efficiency of the immobilized lipase, respect to the free one. In this respect, covalent immobilization with *p*-hydroxybenzaldehyde linker exhibits slightly better behavior that that obtained by physical retention on previously demineralized sepiolite. In this respect, to definitively conclude which is the best immobilization procedure it is necessary to evaluate the biocatalytic behavior after the successive reuse of both immobilized systems.

### 2.2. Reusability of the Different Systems of Immobilized Lipases

The results obtained when conducting successive reactions under standard conditions, using as heterogeneous catalyst systems those obtained by covalent immobilization of the Biolipase-R on sepiolite, activated with the *p*-hydroxybenzaldehyde linker and that obtained after physical entrapment on demineralized sepiolite, respectively, are shown in Table 5 and Table 6. In both cases, immobilization of lipases was carried out at pH 9. Prior to the reuse of the supported enzyme, is was subjected to a wash with a 100 mM phosphate buffer solution at pH 8, and it was dried in a desiccator for 2 h.

**Figure 4:**
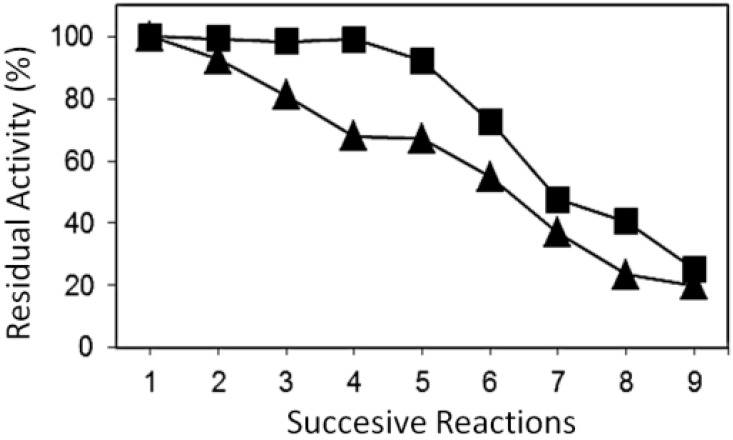
Residual activity of reuses of the two selected immobilization systems of Biolipase R on Sepiolite as support: (■) covalent immobilization obtained by using p-hydroxybenzaldehyde as linker (▲) physical retention of lipase by using demineralized sepiolite obtained after acid treatment.

From the conversion values collected in Table 5 and Table 6 the corresponding values of R_res_ are obtained for the two different immobilized Biolipase R systems. They are shown in Figure 4. It can be clearly seen that the covalent supported lipase system, obtained using *p*-hydroxybenzaldehyde as linker, presents a more stable catalytic behavior, because of it maintains almost 100% residual activity until the fifth use, after which it begins a continuous loss of residual activity with reuse. The supported system obtained by physical retention of Biolipase R using demineralized sepiolite shows on the contrary a progressive loss of activity from the first reuse. In this respect, the results obtained indicated that covalent immobilization with *p*-hydroxybenzaldehyde linker exhibited the best biocatalytic behavior, because the immobilized enzyme showed a remarkable stability as well as an excellent reutilization capacity (more than five successive reuses) without a significant loss of its initial catalytic activity.

The behaviour of both systems is entirely consistent with the method of linking of lipases with the support surface. Thus, the covalent immobilization using *p*-hydroxybenzaldehyde as linker occurs through a chemical linker that irreversibly links the inorganic support and the protein structure of lipases so that it allows formation of a more stable immobilization.

**Table 5:** Conversion (FAEE + MG + DG), selectivity (FAEE + MG) values as well as DG and TG composition, obtained in the successive reuse of Biolipase R covalently immobilized on sepiolite activated with the linker *p*-hydroxybenzaldehyde at pH 9, operating under standard conditions: 30 °C, with 12 mL of sunflower oil, 3.5 mL of absolute ethanol (1:6 oil/ethanol molar ratio) and pH 11, with 1.6 g of supported biocatalyst (15 wt % with respect to the oil used).

N° Reuse	Conversion (%)	Selectivity (%)	DG (%)	TG (%)
1	84.3	59.1	25.2	15.7
2	83.7	53.2	30.5	16.3
3	83.1	52.9	30.2	16.9
4	83.6	54.3	29.3	16.4
5	77.8	42.8	35.0	22.2
6	61.3	36.3	25.0	38.7
7	40.1	28.9	11.2	59.9
8	34.2	22.3	11.9	65.8
9	21.4	15.6	5.8	78.6

**Table 6:** Conversion and selectivity values (in %) obtained in the successive reuse of Biolipase R immobilized by physical adsorption on demineralized sepiolite at pH 9, operating under standard conditions: (30 °C, with 12 mL of sunflower oil, 3.5 mL of absolute ethanol (1:6 oil/ethanol molar ratio) and pH 11, with 1.6 g of supported biocatalyst (15 wt % with respect to the oil used).

N° Reuse	Conversion (%)	Selectivity (%)	DG (%)	TG (%)
1	90.2	60.3	29.9	9.8
2	83.7	58.1	25.6	16.3
3	73.2	43.1	30.1	26.8
4	61.1	37.8	23.3	38.9
5	60.6	32.3	28.3	39.4
6	49.5	29.9	19.6	50.5
7	33.3	20.1	13.7	66.2
8	21.2	16.7	4.5	78.8
9	18.1	12.3	5.8	81.9

However, it is fortuitous that the system that uses *p*-hydroxybenzaldehyde as linker, not only exhibits a more stable linking behaviour, but is also be able to remain efficient after immobilization, retaining 100% of its activity during the first five uses. This is because sometimes the enzyme conformation produced after covalent immobilization of lipases does not allow them to correctly work in the biocatalytic process studied. However, in the current covalent process the conformation obtained was on the contrary especially efficient.

On the other hand, the physical entrapment of lipases into the channel system of demineralized sepiolite is a less stable linkage, progressively losing activity along successive reactions, probably due to the leaching of the enzymes, as it was verified by the presence of proteins in the reaction products (positive Biuret test), which does not occur in the covalent system, where the decline in activity can be attributed to an enzyme deactivation. However, these lipases work in a similar way to those operating in free solutions.

**Figure 5:**
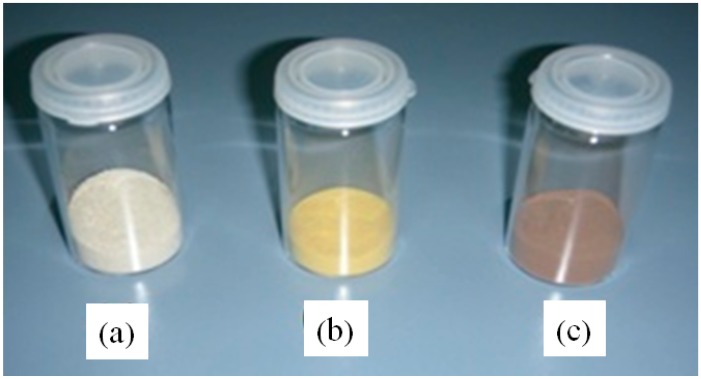
Color changes in the activated support after fixing of the organic molecules constituting the linker which allows the covalent attachment of lipases. From left to right are presented some pictures of (**a**) sepiolite/AlPO_4_ support; (**b**) sepiolite/AlPO_4_ functionalized support after microwave heating with 4-aminobenzylamine; (**c**) functionalized support with terephtaldialdehyde.

Finally, after comparing the results obtained with the current biocatalysts, achieved by immobilization of Biolipase R on sepiolite, with those previously achieved with commercial immobilized lipase, Lipozyme RM IM [34,47], it can be concluded that although they all exhibit high initial conversion values, Lipozyme RM IM exhibited a behavior very similar to that obtained with Biolipase R physically entrapped inside the channels of the demineralized sepiolite (Figure 5). Similar behaviour was also obtained with PPL entrapped in sepiolite [22]. Thus, the deactivation curve along the successive reuses presented a similar profile, with a remarkable continued decreased in activity along the uses since the beginning of its utilization. This behavior may be explained by the fact that in the commercial biocatalyst Lipozyme RM IM [34,47], a lipase from *Rhyzopus oryzae*, is similarly retained by physical adsoption on an ion exchange resin.

The covalent immobilization of PPL using the same described here methodologies allowed a higher number of reuses [24,25], but the price and availability of the PPL compared with those obtained from TLL, in its commercial form Lipopan [26] or ROL as Biolipase R [33], makes them less competitive than the biocatalyst obtained by covalent immobilization of Biolipase R on inorganic supports like sepiolite, following the solid-phase synthesis here applied. Therefore, of all the lipases so far investigated, PPL [22,23,24,25], TLL [26], RM IM [34,47] and the ROL [33], the best biocatalyst identified to carry out the 1,3 selective ethanolysis of sunflower oil, is obtained by covalent immobilization of ROL, from commercial lipase Biolipase R, on sepiolite, a low cost support of high surface area.

## 3. Experimental Section

### 3.1. Support Activation and Functionalization of Sepiolite for Covalent Lipase Immobilization

The sepiolite used as support (Tolsa S.A, Zaragoza, Spain) is a cheap natural silicate with a high surface that presents a fibrous structure (Figure 4). The theoretical formula of the unit cell is Si_12_O_30_Mg_8_(OH)_6_(H_2_O)_4_·8H_2_O, where the Si^4+^ and the Mg^2+^ can be partially substituted by Al^3+^, Fe^2+^ and alkaline ions. Each Mg atom completes its coordination with two molecules of water. This inorganic solid cannot be used directly for the immobilization of lipases. Therefore, it is necessary to proceed to its activation, before its organic functionalizacion, in order to chemically link a protein structure, as is the case of lipases.

In this way, the sepiolite was subjected to a surface activation process, through a soft precipitation as a gel of AlPO_4_ to obtain a final Sepiolite/AlPO_4_ ratio of 80/20. The synthesis of AlPO_4_ was carried out according to a previously described sol–gel method [35,36,37,45,46,47]. This amorphous material AlPO_4_ (Al/P = 1) was obtained by precipitation from aqueous solutions of AlCl_3_·6H_2_O and H_3_PO_4_ (85 wt %) at pH = 6.1 by addition of ammonium hydroxide. The solid obtained after filtration was then washed with isopropyl alcohol and dried at 120 °C for 24 h. In the present case, the resulting powder was calcined by heating at 350 °C for 3 h and then screened to a particle size <0.149 mm (100 mesh size) before its use as activated support for anchoring of a functionalized linker with pendant benzaldehyde through the reaction of appropriate molecules with the surface –OH groups of inorganic solids. This amorphous solid with high surface area and high density of Bronsted acid sites, is particularly suitable for the surface functionalization with some organic molecules, through the reaction of the surface acid sites with amino or hydroxyl groups of these activating molecules [35,36,37,45,46,47].

Thus, the functionalization of the activated AlPO_4_/sepiolite system, used as inorganic support for covalent enzyme immobilization [29,33,34,37], is performed by forming two “linkers” with adequate functionalization in order to the enzyme can be fixed to the support by a covalent bond. In this respect, are reacted the Bronsted acid sites of the support surface with an organic linker provided with a pendant aldehyde, which with the free amino groups of the enzyme forms an imine bond. According to solid phase stepwise synthesis, collected in Figure 1 and Figure 2, the functionalization reactions are carried out in conventional microwave oven, in the absence of solvent. In this way organic-inorganic hybrids capable of covalently fixing lipases to the surface of an inorganic support, are obtained in a fast and clean way. In these successive treatments functionalization with superficial aldehyde groups, which are able to immobilize enzymes by reaction with the ε-lysine residues of lipase proteins were obtained in both cases. Due to the increased electrophilic character of benzaldehyde, the aromatic Schiff’s-base obtained seems to be more stable than those usually obtained by using glutaraldehyde [39,40,41] or other aliphatic aldehydes like glycidol [39,40,41,42,43,44].

Regarding the functionalization of the support by using *p*-hydroxybenzaldehyde, according to the reaction scheme shown in Figure 1, it is developed a process in which the phenolic hydroxyl group of the *p*-hydroxybenzaldehyde reacts with a Bronsted acid group on the solid surface. In this process a phosphoester bond which links a benzaldehyde to the inorganic solid surface, and the removal of a water molecule occur. Thus, according to the stepwise synthesis methodology [35,36,37], illustrated in Figure 1, the functionalization of the support takes place through the reaction between 20 g of activated support and 2 g of *p*-hydroxybenzaldehyde in a conventional microwave oven for 15 minutes at 380 W, in periods of 2 min. In this respect, first of all the solid was homogenized with *p*-hydroxybenzaldehyde for one hour in a rotary evaporator at room temperature These amounts provide the conditions-called “incipient wetness”, which allow the homogenization of the mixture employing the minimum amount of solvent. After this period, the ethyl ether (from Panreac, Barcelona, Spain) is removed by heating in water bath in the same rotary evaporator without applying vacuum, and then the activation by microwave is carried out.

For covalent immobilization of the lipase with a longer linker (Figure 2), following a previously described methodology [35,36,37,45,46,47], the organic linker is built by the formation of phosphamides bonds, through the reaction of Bronsted acid surface groups of the AlPO_4_/sepiolite support, with the diamine 4-aminobenzylamine (Step 1), subsequently followed by its reaction with an aromatic dialdehyde, the terephtaldicarboxaldehyde (Step 2), that provided a terminal carbonyl group. Then, (Step 3) these aromatic aldehydes could react with the free amino groups of the lysine groups of lipases, to form imine bonds, thus achieving the covalent immobilization of the enzyme through the ε-amino group of lysine residues. The functionalization process is carried out, therefore, in two stages, and can be performed in the same reaction flask after a suitable wash of the solid after each reaction. Moreover, both reactions are carried out in a fast and clean way, in a conventional microwave oven.

The reaction of the surface acid groups of the support, with one of the terminal amino groups of the amino benzylamine, is carried out by the homogenization in a 100 mL flask, of 20 g of the AlPO_4_/sepiolite support, 80 mL of ethyl ether and 4 mL of aminobenzylamine (99%, Merck, Darmstadt, Germany), for one hour in a rotary evaporator at room temperature, under so-called “incipient wetness” conditions. Then diethyl ether is removed by heating in a water bath in the same rotary evaporator but without applying vacuum, since it boils at 33 °C. Then the flask is placed in a domestic microwave oven, where it is subjected during fifteen minutes to 380 W irradiation (in 2 min periods). After several washes of the functionalized solid with ethyl ether to remove the reagent that it hasn’t reacted with the solid, this is impregnated again in a rotary evaporator with terephthalic aldehyde (4 g), which after the removal of ethyl ether in a rotary evaporator, is subjected to a new microwave activation, irradiating at 380 W for five minutes. Once the unreacted terephthalic aldehyde is removed by washing with ethyl ether, the activated support is ready for use in the immobilization of lipases. Changes produced on the surface of the inorganic solid used as support in the subsequent treatments that lead to functionalization with surface aldehyde groups (Figure 2) which are able to react with the ε-amino groups of lysine residues of lipases can be evidenced. These changes due to the high conjugation of the molecule bound to the inorganic support, after the formation of the imine bonds can also be visualized in Figure 5.

### 3.2. Demineralized Sepiolite System Used for Physical Retention of Lipases

The acid demineralization treatment of the natural sepiolite was carried out under the previously described conditions [22]. Each eight hours, the presence of Mg was determined in the supernatant using “titan yellow” as a specific indicator. The acid attack was repeated until the absence of Mg in the filtrate was determined. To maintain the thus obtained fibrous structure, the resulting solid was maintained all the time under incipient wetness conditions [35,37].

### 3.3. Immobilization of Biolipase R on Different Sepiolite Activated Supports

The immobilization of Biolipase R was carried out at room temperature by introducing the functionalized inorganic solid (0.5 g) with the ROL (0.04 g) in a reaction flask (50 mL) with 6 mL of ethanol, soft mechanical stirring and refrigeration for 24 h, stirring occasionally every three to four hours to facilitate the covalent interaction of the ε-amino group of lysine residues of the ROL with the aldehyde groups of linkers (Figure 1 and Figure 2). Finally, prior to its use, ethanol (6 mL) was added to the mixture and the solid with the immobilized lipase was then separated by filtration and centrifugation from the solution containing the remaining non-immobilized lipase. The catalytic activity of this dissolution was proportional to the amount of dissolved Biolipase R. Thus, we could easily determine the amount of lipase, which had not been immobilized, and was hence remaining in supernatant dissolution. The comparison of this value with the activity of immobilized and free Biolipase R enzymes would allow us to determine the amount of immobilized enzyme and its efficiency [35,36,37,45,46,47].

In order to evaluate the subsequent effect of demineralization treatment, a series of tests were made in which Tolsa S.A. sepiolite from Vallecas (Madrid) with only a wash with deionized water was employed. These results were compared with those obtained when the same sepiolite is subjected to a demineralizing acid treatment, as lipase support [22], for the production of biodiesel by transesterification of sunflower oil with absolute ethanol (Table 3 and Table 4). To perform the immobilization of the enzyme on natural sepiolite, after and before demineralization treatment, the same methodology in which the biocatalysts with covalent immobilization are obtained is followed [22].

### 3.4. Alcoholysis Reactions

The alcoholysis reactions were performed in a 50 mL round bottom flask under continuous magnetic stirring at controlled temperature (30 °C) and at a pH value near to 11, as measured by indicator paper color change. This pH environment was achieved by adding a very small amount of aqueous 10 N NaOH solution. The reaction mixture was comprised of 9.4 g (12 mL, 0.01 mol) sunflower oil, 3.5 mL of absolute ethanol (1/6 oil/ethanol volume ratio) and 12.5 μL of 10 N NaOH. Reaction mixtures were stirred with a conventional magnetic stirrer at a stirring speed higher than 300 rpm, to avoid mass transfer limitations, for a reaction time of 2 h. These conditions, obtained evaluating the enzyme in the free form [33], were determined as optimal for this lipase. As biocatalyst, 0.5 g of solid containing 0.01 g immobilized Biolipase R is applied. Free ROL (0.01 g) was also used as reference to determine the efficiency and amount of immobilized enzyme [33]. The amount of immobilized enzyme used in both cases of immobilization covalent and physical respectively, was 15% by weight with respect to the oil used (Table 5, Figure 4).

The influence of pH values were evaluated by adding different volumes (12.5–50 μL) of 10 N NaOH aqueous solutions. In this regard, a blank reaction in the presence of the highest quantity of NaOH solution was performed to rule out a potential contribution from the homogeneous NaOH catalyzed reaction. Less than 10% conversion of the starting material was obtained, so any homogenous base catalysis contribution can be considered negligible under the investigated conditions.

### 3.5. Analytical Method

Reaction products were monitored by capillary column gas chromatography, using a Varian 430- GC gas chromatograph, connected to a HT5 capillary column (25 m × 0.32 mm ID × 0.1 μm, SGE, Supelco) with a flame ionization detector (FID) at 450 °C and splitless injection at 350 °C. Helium is used as carrier gas, with a flow of 1.5 mL/min. It has been applied a heating ramp from 90 °C to 200 °C at a rate of 7 °C/min, followed by another ramp from 200 °C to 360 °C at a rate of 15 °C/min, maintaining the temperature of the oven at 360 °C for 10 min using as internal standard *n*-hexadecane (cetane) to quantify the content of ethyl esters and the different glycerides (mono-, di- and triglycerides) with the help of several commercial standard fatty acid esters. This method allows us to completely analyze the sample in a single injection and in a time not exceeding 60 min, which simplifies the process and increases the speed of analysis [24,25,26,33,34].

Considering that sunflower oil is constituted by a mixture of fatty acids in variable proportion (mainly linoleic, oleic, palmitic and stearic acids), the reaction results are expressed as the relative amounts of the corresponding ethyl esters (Fatty Acid Ethyl Esters—FAEE,), monoglycerides (MG) and diglycerides (DG) that are integrated in the chromatogram. The amount of triglycerides (TG) which has not reacted is calculated from the difference to the internal standard (cetane). Thus, the Conversion includes the total amount of triglyceride transformed (FAEE + MG + DG) in the ethanolysis process and the Selectivity makes reference to the relative amount of FAEE + MG obtained. The latter are those having retention times close to the cetane standard, which is the reference hydrocarbon for diesel fuel [25].

### 3.6. Evaluation of the Efficiency of Different Lipase Immobilization Procedures

The efficiency of the immobilization processes of the different supported lipase systems synthesized, is obtained from the comparison of the transesterification reaction rates obtained with the free lipase, in native form, as well as with the separated supernatant after the immobilization process, and with the heterogenized lipase system. In this way, the amount of enzyme retained by immobilization can be known by calculating the percentage of immobilized enzyme, E_imm_, which is calculated by the difference between the catalytic activity of the free native enzyme, r_nat_ and the activity of the supernatant separated by centrifugation in the immobilization processes, r_filt_ (Equation (1)):

(1) $$\left[E_{imm}=\frac{r_{nat}-r_{filt}}{r_{nat}}x100\right]$$

The residual activity after immobilization is obtained from the percentage of catalytically active immobilized enzyme, E_res_, it is determined from the relationship between the activities of the free native and immobilized enzyme (Equation (2)):

(2) $$\left[E_{res}=\frac{r_{imm}}{r_{nat}}x100\right]$$

The specific activity, E_spe_, is indicative of the efficiency of the immobilized enzyme with respect to the free native enzyme, and it can be obtained from the relationship (Equation (3)):

(3) $$\left[E_{spe}=\frac{E_{res}}{E_{imm}}x100=\;\frac{r_{imm}}{r_{nat}-r_{fil}}x100\right]$$

### 3.7. Evaluation of the Efficiency of Reuse of the Immobilized Systems

The efficiency of every lipase immobilized system is evaluated from the residual activity of reuses, R_res_. This parameter is obtained, from the percentage of the ratio between the biocatalytic activity of the supported lipase system after a number N of reuses and the activity obtained in the first use (Equation (4)):

(4) $$\left[R_{res}=\frac{Conv_{N}}{Conv_{1}}\times 100\right]$$

## 4. Conclusions

The results achieved regarding to the functionalization of the support for the covalent immobilization of the lipases by using two different organic linkers, are in both cases satisfactory, although they exhibit some differences showing the importance of the pH conditions when the Biolipase R immobilization process is developed. It can be concluded therefore that the pH value applied in the immobilization process of lipases is extremely important, according to the important differences obtained in the E_res_ values at the studied pH values, 7 and 9, respectively. However, in both cases the residual activity at pH 9 is higher than that obtained at pH 7. This resultant residual activity, which is due to the contribution of two different parameters—the percentage of immobilized active lipase (E_imm_) and the efficiency of such immobilized lipases (E_esp_)—is in this way decisively influenced to a different extent by the method of immobilization (covalent or physical), the kind of linker and also by the pH conditions under which the immobilization process takes place.

In summary, the comparison between both organic linkers able to carry out the surface activation of inorganic solids seems to recommend the use of *p*-hydroxybenzaldehyde as activating agent, operating at pH 9. Furthermore, this method is comparatively simpler, because the activation is carried out in only one step, compared to the two steps required with the process that uses 4-aminobenzylamine plus terephthalic aldehyde.

On the other hand, the covalent immobilisation affects more intensely these two parameters (E_imm_ and E_esp_) than the physical entrapment on demineralized sepiolite. However, the combination of both parameters in E_res_ leads to results that are quite similar with both types of immobilization, for the first reaction, with a slight advantage of the covalent linker system, using *p*-hydroxybenzaldehyde, respect to the physical immobilization on demineralized sepiolite. Nevertheless, covalent immobilization ensures a greater advantage in terms of reuses, because the covalently immobilized lipases showed a remarkable stability as well as an excellent capacity of reutilization (more than five successive reuses) without a significant loss of the initial catalytic activity. Consequently, the obtained results let us conclude that covalent immobilization of Biolipase R on sepiolite, by using *p*-hydroxybenzaldehyde as organic linker, is the best option for its use as heterogeneous biocatalyst in biofuel production by an enzymatic method, that could allow a more efficient fabrication of biodiesel minimizing the production of glycerol as a waste by-product.
